# Algal polysaccharides–Selenium nanoparticles regulate the uptake and distribution of selenium in rice plants

**DOI:** 10.3389/fpls.2023.1135080

**Published:** 2023-03-10

**Authors:** Chunmei Yang, Chaoxin Wang, Zaid Khan, Songpo Duan, Zhiming Li, Hong Shen

**Affiliations:** College of Natural Resources and Environment, South China Agricultural University, Guangzhou, China

**Keywords:** rice plants, APS-SeNPs, absorb, accumulation, selenate, selenite

## Abstract

**Introduction:**

Selenium (Se) is an essential trace element required for proper human and animal health.

**Methods:**

In this paper, we investigated the uptake and distribution characteristics of a new Se fertilizer, which comprises algal polysaccharides–selenium nanoparticles (APS-SeNPs), in rice plants in both hydroponic and pot experiments.

**Results:**

The results from the hydroponic experiments revealed that the rice root uptake of APS-SeNPs fitted the Michaelis–Menten equation, with a *V*_max_ of 13.54 μg g^−1^ root dry weight (DW) per hour, which was 7.69 and 2.23 times those of selenite and selenate treatments, respectively. The root uptake of APS-SeNPs was inhibited by AgNO_3_ (64.81%–79.09%) and carbonyl cyanide 3-chlorophenylhydrazone (CCCP; 19.83%–29.03%), indicating that the uptake of APS-SeNPs by rice roots is mainly *via* aquaporins and is also affected by metabolic activity. Moreover, sulfur deficiency caused rice roots to absorb more APS-SeNPs, but treatment with APS-SeNPs increased the expression of the sulfate transporter *OsSULTR1;2* in the roots, suggesting that *OsSULTR1;2* is probably involved in the uptake of APS-SeNPs. The application of APS-SeNPs significantly increased the Se content in rice plants and the apparent Se uptake efficiency compared with selenate and selenite treatments. Most of the Se in the roots of rice plants was distributed in the cell wall, while it was primarily located in the cytosol in the shoots when treated with APS-SeNPs. The results from the pot experiments indicated that the application of Se enhanced the Se content of each rice tissue. It is worth noting that the Se content in brown rice under APS-SeNP treatment was higher than that under selenite or selenate treatment and was mainly concentrated in the embryo end, with the Se in organic form.

**Discussion:**

Our findings provide important insights into the uptake mechanism and the distribution of APS-SeNPs in rice plants.

## 1 Introduction

Selenium (Se) is an essential trace element that is an important component of many selenoproteins and selenoenzymes in mammals. It has a variety of biological functions, such as antioxidant activity and immunity enhancement, and a role in delayed aging (Wang et al., 2020a; Wang et al., 2022). However, the human body cannot synthesize Se and needs to obtain it from external sources. Plants, especially cereal crop species such as rice and wheat, are the main sources of dietary Se for humans (Zhou et al., 2020; Zhang and Cai, 2022). Under natural conditions, the low amount of Se in the soil can be absorbed by plants, but it is difficult to meet an individual’s daily need for Se supplementation (Li et al., 2017; Zhang and Cai, 2022). The production of Se-enriched crops by agronomic biofortification has been implemented in many regions around the world. Nevertheless, the uptake of Se by plants can be affected by the form of Se, the type of Se fertilizer, and the external environment. Therefore, exploring the mechanisms of Se uptake by plants is important for increasing the Se content of plants and improving the Se nutrition of humans.

Se exists in nature either in the form of selenate, selenite, or elemental Se. Selenate and selenite are the major forms found in the soil (Yuan et al., 2022), but their safety range is narrow. Selenium nanoparticles (SeNPs) are bright red nanoparticles formed by the biotic or abiotic reduction of Se and are characterized by low toxicity and high biological activity (El-Badri et al., 2022). Li et al. (2020) showed that the toxicity order of Se to garlic was selenate > selenite > SeNPs. The uptake and transport of Se by plants are closely related to its chemical form. Since selenate is chemically similar to sulfate, it is taken up through high-affinity sulfate transporters in the roots and is rapidly translocated into the shoots *via* an energy-dependent process. Selenite enters the plant roots through a phosphate or silicate transporter, and it is mostly converted to organic Se and accumulated in the roots, the process of which is also energy-dependent (Sors et al., 2005). In contrast, the uptake mechanism of SeNPs by plants is not clear. It has been suggested that only SeNPs with diameters smaller than the pore size of the cell wall can reach the plasma membrane, but the pore size of the cell wall is only a few nanometers (Dietz and Herth, 2011). Wang et al. (2020a) showed that plant roots can absorb SeNPs with an average diameter of 93 nm and that aquaporin inhibitors affect plants’ uptake of SeNPs. In addition, a hydroponic experiment showed that the particle size of SeNPs did not affect their uptake (Wang et al., 2019b), while the study by Lyu et al. (2022) showed that the conversion rate of large SeNPs in the soil was significantly higher than that of smaller SeNPs. Nevertheless, there are many other debates and uncertainties about the uptake mechanism of SeNPs by plants, and further research at the physiological and molecular levels is needed.

SeNPs have unique physical, chemical, and biological properties; however, their stability is poor, and they tend to easily aggregate into gray and black Se. Therefore, dispersers or stabilizers need to be added for fixation (Jia et al., 2015). Algal polysaccharides are a class of natural polymers containing a large number of active hydroxyl groups with good cellular affinity and compatibility, and they constitute a potential class of biological resources for building nanomaterials (Li et al., 2022). Algal polysaccharides can significantly improve the stability and biocompatibility of SeNPs. Recent studies have shown that SeNPs combined with polysaccharides improved the free radical scavenging ability and showed stronger antioxidant activity (Cao et al., 2021). Tang et al. (2021) showed that *Gracilaria lemaneiformis* polysaccharides–selenium nanoparticles (GLPs-SeNPs) had excellent biocompatibility. Furthermore, Zhao et al. (2022) showed that *Sargassum fusiforme* (a type of algae) polysaccharides–selenium nanoparticles (SFPS-SeNPs) can reduce the toxicity of Se, and SFPS-SeNPs improved the stability of SeNPs and exerted the biological activities of SFPS (Wang et al., 2021b). However, there are no studies on APS-SeNPs in the field of agricultural Se-enriched fertilizers. The distribution of APS-SeNPs in each organ of rice is still unclear.

Rice is a staple food for nearly half of the world’s population (Wang et al., 2022). According to the Food and Agriculture Organization of the United Nations (FAO), the world’s annual rice production was 512.8 million tons in 2022. Rice is a major source of Se supplement for the human body. However, rice is a non-Se-rich plant species whose grains have a low Se content; therefore, Se-enriched rice crops need to be produced through agronomic biofortification, among other means. The low toxicity and high bioavailability, among the other excellent physiological properties of APS-SeNPs, which constitute a new type of Se-enriched fertilizer, have received widespread attention from researchers, but it is not clear how APS-SeNPs are absorbed by plant roots and how they are transported and distributed in different plants parts. Therefore, in this study, APS-SeNPs, selenite, and selenate were used as Se sources in hydroponic and pot experiments, which aimed 1) to examine the uptake characteristics of APS-SeNPs; 2) to investigate the uptake channels of APS-SeNPs; and 3) to compare the uptake, translocation, and accumulation distribution characteristics of selenate, selenite, and APS-SeNPs in rice given short-term (24 h in hydroponic cultivation) and long-term (grown in pots until harvest) treatments. The results of this study will contribute to a more in-depth understanding of the uptake and translocation of APS-SeNPs in rice and will provide new insights into the potential application of APS-SeNPs in agricultural production.

## 2 Materials and methods

### 2.1 Rice seeding cultivation

Rice seeds (Luodao 998) were surface sterilized in 30% (*v*/*v*) H_2_O_2_ for 15 min, washed three times with deionized water, and germinated in Petri dishes at 25°C in the dark. After germinating for 7 days, the rice seedlings were grown in plastic containers with half-strength Kimura nutrient solution (pH 5.5–6.0) with the following composition (in micromoles per liter): 91.5 KNO_3_, 91.0 KH_2_PO_4_, 183.0 Ca(NO_3_)_2_·4H_2_O, 273.5 MgSO_4_·7H_2_O, 182.5 (NH_4_)_2_SO_4_, 40.0 Fe(Ш)–EDTA, 0.4 ZnSO_4_·7H_2_O, 3.0 H_3_BO_3_, 1.0 (NH_4_)_6_Mo_7_O_24_·4H_2_O, 0.5 MnCl_2_·4H_2_O, and 0.2 CuSO_4_·5H_2_O. The solution was replenished every 3 days.

### 2.2 APS-SeNP preparation and characterization

Chemical APS-SeNPs were synthesized according to Tang et al. (2021), with some modifications. APS-SeNPs were synthesized using APS as a stabilizer, and sodium selenite was reduced using ascorbic acid. The synthesized solution was dialyzed in the dark at 4°C for 48 h to remove superfluous ascorbic acid and sodium selenite. Finally, the prepared APS-SeNPs were collected *via* centrifugation and resuspension in deionized water. The morphology and microstructure of APS-SeNPs were observed with field emission scanning electron microscopy (SEM) (Verios 460; FEI, Hillsboro, OR, USA) and transmission electron microscopy (TEM) (Talos F200S; FEI, Hillsboro, OR, USA). The element composition of APS-SeNPs was analyzed using energy-dispersive X-ray spectroscopy (EDS) (30p; Thermo Fisher Scientific, Waltham, MA, USA) in combination with TEM (Talos F200S; FEI, Hillsboro, OR, USA). In addition, the particle size and zeta potential were measured using a laser particle size analyzer (Zatasizer Nano ZS 90, Malvern, UK).

Analytical-grade sodium selenate (Na_2_SeO_4_) and selenite (Na_2_SeO_3_) were purchased from Chengdu Ekeda Chemical Reagent Company (Chengdu, China) and used as the selenate and selenite sources, respectively.

### 2.3 Hydroponic experiment

#### 2.3.1 Uptake characteristics of APS-SeNPs

##### 2.3.1.1 Concentration uptake kinetics

Rice seedlings exhibiting the same growth for 45 days were selected for treatment. The treatment solution comprised a series of concentrations ranging from 0 to 20 μmol L^−1^ (0, 1, 5, 10, 15, and 20) of APS-SeNPs, selenite, or selenate. The rice roots were harvested after 1 h. The roots were then soaked in a desorption solution (1 mM CaSO_4_ and 2 mM MES) for 15 min, washed with deionized water three times, and dried with an absorbent paper. The material was dried, powdered, and stored in bags for subsequent Se content-related experiments.

##### 2.3.1.2 Time uptake kinetics

The 45-day-old uniform rice seedlings were transferred into a 400-ml container for further treatment. The seedlings were treated with a solution of 10 μmol L^−1^ APS-SeNPs, selenite, or selenate, and the rice roots were collected after 1, 6, 12, 24, and 36 h.

#### 2.3.2 Uptake channel of APS-SeNPs

##### 2.3.2.1 Response of inhibitors to different concentrations of APS-SeNP uptake by rice

According to the method of Wang et al. (2020a), with some modifications, 45-day-old uniform rice seedlings were transferred into 400 ml containers and their roots were harvested after 1 h of treatment. A treatment solution consisting of 10 or 50 μmol L^−1^ APS-SeNPs plus different inhibitors [10 μmol L^−1^ carbonyl cyanide 3-chlorophenylhydrazone (CCCP)/0.1 mM AgNO_3_/0.1 mM 4,4-diisothiocyanatostilbene-2,2-disulfonic acid disodium salt hydrate (DIDS)] and a control treatment solution (no inhibitor) were applied at the same time. AgNO_3_ is an aquaporin inhibitor, DIDS is an anionic inhibitor, and CCCP is a respiratory inhibitor, which was dissolved in ethanol, with the final ethanol concentration being 0.01% (*v*/*v*). Thus, 10 or 50 μmol L^−1^ APS-SeNPs with 0.01% (*v*/*v*) ethanol was added as the control.

##### 2.3.2.2 Effect of phosphorus/sulfur nutrition on APS-SeNP uptake by rice

In reference to the method of Wang et al. (2020b), with some modifications, the 45-day-old uniform rice seedlings were transferred into normal, phosphorus (P)- or sulfur (S)-deficient (in which phosphate or sulfate was replaced by their chloride counterparts), and S- or P-supplemented [with an additional 1 mM (NH_4_)_2_SO_4_ or KH_2_PO_4_] nutrient solutions and were pretreated for 5 days. Subsequently, the seedlings were transferred into the normal nutrient solution plus 10, 25, or 50 μmol L^−1^ APS-SeNPs, after which the roots were harvested after exposure for 24 h.

##### 2.3.2.3 Relative gene expression analysis

The 45-day-old uniform rice seedlings were transferred into the nutrient solution plus 0, 10, or 50 μmol L^−1^ APS-SeNPs. The roots were then harvested after exposure for 1, 12, and 24 h, frozen in liquid nitrogen, and stored at −80°C for gene expression analysis.

#### 2.3.3 Transport and accumulative distribution of APS-SeNPs in rice

The 45-day-old uniform rice seedlings were transferred into a 400-ml container for treatment. Thereafter, 200 ml of a nutrient solution containing 10 μmol L^−1^ APS-SeNPs, selenite, or selenate was added to each vessel, and the rice plants were harvested after exposure for 24 h. The roots and shoots were separated, frozen in liquid nitrogen, and stored at −80°C for analysis of the Se contents in the subcellular fractions. Afterward, the roots and shoots were separated, dried, crushed into a powder, and weighed before the Se content was measured. Each treatment was replicated three times.

### 2.4 Pot experiment

A pot experiment was conducted in the greenhouse of South China Agricultural University in Guangzhou, Guangdong Province, China (113°21′ E, 23°9′ N). The soil was air-dried and passed through a 2.0-mm sieve. The basic physicochemical analysis results of the test soil were as follows: 0.25 mg kg^−1^ total Se, 20.49 g kg^−1^ organic matter, 129.43 mg kg^−1^ alkaline hydrolyzable nitrogen, 30.71 mg kg^−1^ available phosphorus, and 89.92 mg kg^−1^ available potassium, pH 6.15. All the physicochemical properties of the soil were determined according to the protocols described by Bao (2000). Each pot (30 cm in height and 20 cm in diameter) was filled with 10 kg of the test soil, 5.6 g compound fertilizer (N/P_2_O_5_/K_2_O = 24:8:20), and 0.5 mg kg^−1^ APS-SeNPs, selenite, or selenate (Se was prepared into a solution and sprayed evenly into the soil, with the soil sieved through a 2.0-mm sieve 2 days after spraying to ensure even distribution of Se). In addition, 30-day-old uniform rice seedlings were transferred into plastic pots, with three plants planted in each pot. Management of the experiment was carried out daily during the plant growth period. After harvesting the rice, the roots, stems, leaves, and grains were dried, crushed, and subjected to measurements of the Se content.

### 2.5 Flowchart of the study

The study flowchart is shown below.

**FLOWCHART:**
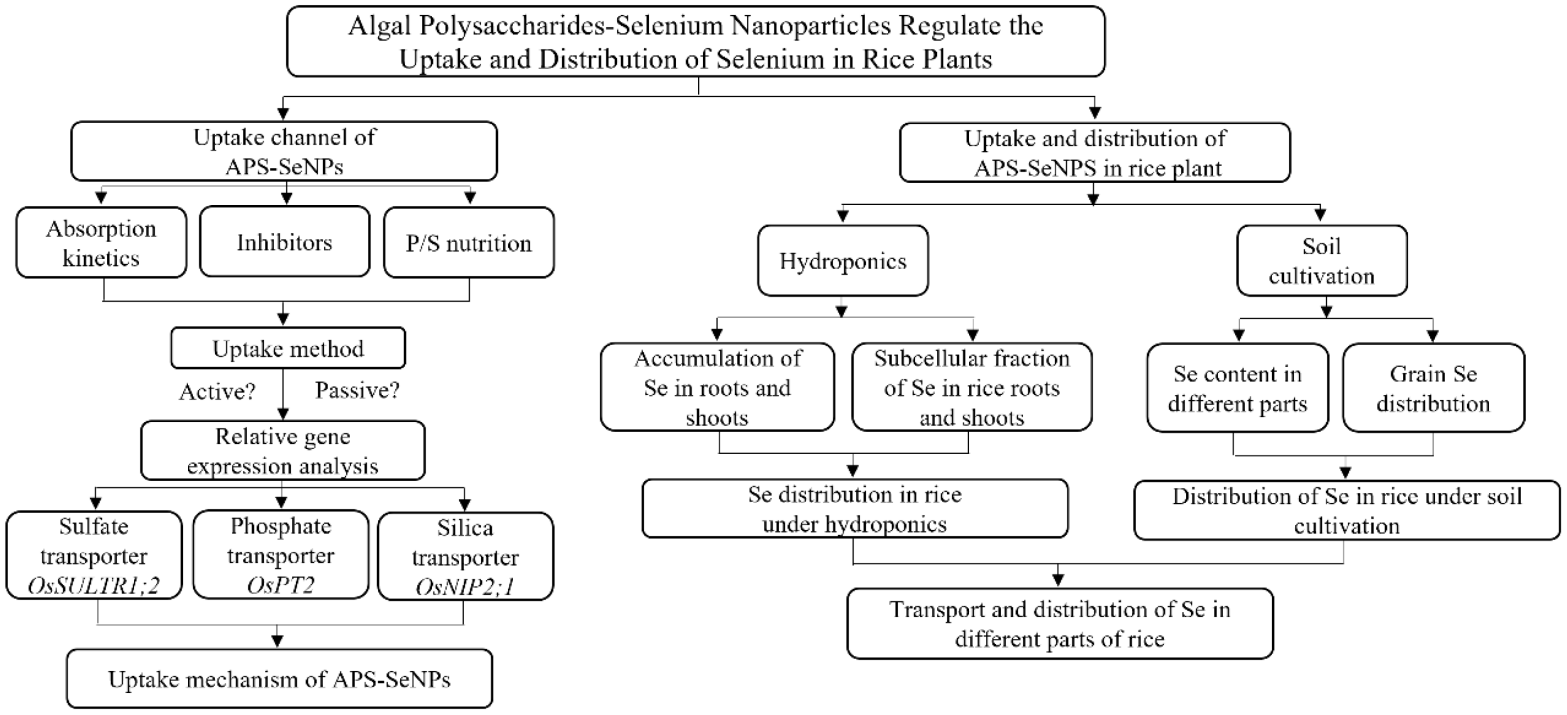


### 2.6 Determination index and methods

#### 2.6.1 Determination of Se concentrations

##### 2.6.1.1 Analysis of total Se

The total Se content was determined using a hydride atomic fluorescence spectrometer (AFS-922; Beijing Jitian Instrument Company, Beijing, China) according to the methods of Zhang et al. (2019) and Di et al. (2023). The samples (0.25–0.50 g) were soaked overnight at room temperature with 10 ml HNO_3_–HCIO_4_, digested to 2 ml at 150°C, cooled, and were added to 5 ml (6 M) HCI. Heating was continued until the solution became clear and colorless, with the appearance of white smoke. The Se content per sample was then determined.

##### 2.6.1.2 Organic Se

In reference to the methods of Deng et al. (2017), with some modifications, 2.5 g samples were weighed, 20 ml HCl (6 M) was added, and the resulting mixture was shaken at 60°C and 200 rpm for 18 h. The supernatant was then collected and transferred into a separatory funnel, 5 ml cyclohexane was added for extraction, and the aqueous phase was collected. Of the aqueous phase, 4 ml was pipetted into a test tube, boiled for 20 min, cooled, and brought to 10 ml with ultrapure water. The inorganic Se content was determined using a hydride atomic fluorescence spectrometer. The organic Se content was calculated by taking the total Se content minus the inorganic Se content.

#### 2.6.2 Relative gene expression analysis

According to the method of Santaniello et al. (2017), with some modifications, the messenger RNA (mRNA) of rice roots was extracted using a column-based total plant RNA extraction and purification kit (B518661-0100; Sangon Biotech, Shanghai, China). To check for RNA integrity, 1% agarose gel electrophoresis was performed on all the samples, followed by spectrophotometric quantification. Subsequently, RNA was reverse transcribed using a HiScript III 1st Strand cDNA Synthesis Kit (R323-01; Vazyme, Nanjing, China), which contained a genomic DNA (gDNA) eraser. The sulfate transporter *OsSULTR1;2*, the phosphate transporter *OsPT2*, and the silicate transporter *OsNIP2;1* were analyzed in the roots of rice plants using a Real-Time PCR Detection System (ABI7500; Applied Biosystems, Carlsbad, CA, USA). The primers were in reference to Liang et al. (2019) and were synthesized by the staff at Sangon Biotech Company, as shown in **Table 1**. *ACTIN* (Os03g0718100) was used as an internal control to normalize the gene expression data. The 2^−ΔΔCt^ method was used to calculate the expression levels of the target genes.

**Table 1:** Primer sequences used in this study.

Gene	Primer sequences
*ACTIN*(Os03g0718100)	FW: TCCATCTTGGCATCTCTCAG
	RV: GTACCCGCATCAGGCATCTG
*OsPT2*(Os03g05640)	FW: AAACTTCCTCGGTATGCTCATG
	RV: ATGTTTATGACATCACGCTTGG
*OsNIP2;1*(Os02g51110)	FW: AACATCCAAGTGTGATAGGACG
	RV: ACACAAAGACGTAGCTAGTGAT
*OsSULTR1;2*(Os03g0195500)	FW: TCAAAGAAGAACCCGCTAGATT
	RV: GCAATTCCAAGGAAGCCTTTAA

FW, forward; RV, reverse

#### 2.6.3 Determination of the Se contents in subcellular fractions

The Se contents in the subcellular fractions of rice tissues were determined as described by Wang et al. (2021a), with some modifications. Fresh samples (0.4 g) were accurately weighed and ground in 10 ml of pre-cooled extraction buffer (1 mM dithioerythritol, 250 mM sucrose, and 50 mM Tris–HCl, pH 7.5) and the homogenate was taken. The homogenate was then centrifuged at 300 × *g* for 10 min, with the residue representing the cell wall fraction (F1), while the supernatant was centrifuged at 20,000 × *g* for 30 min, with the residue representing the organelle fraction (F2) and the supernatant constituting the soluble cytosol fraction (F3). All of these processes were performed at 4°C. Finally, the different fractions were digested and the Se content was measured.

#### 2.6.4 Spatial distribution of Se in rice grains

Referring to the methods of Zhang et al. (2020a), after harvesting the rice grains, some of the brown grains were cut into three equal sections: the embryo end, the middle, and the non-embryo end. Thereafter, the materials were dried and the Se content of each section was measured. The other parts of the brown grains were cut into two lengths with a blade and then dried in an oven at 40°C using TEM (Talos F200S; FEI, Hillsboro, OR, USA) in combination with EDS (30p; Thermo Fisher Scientific, Waltham, MA, USA). Subsequently, the X-ray fluorescence images of carbon (C), oxygen (O), and Se elements were examined.

### 2.7 Statistical analysis

The Se uptake kinetics was described based on the Michaelis-Menten equation, as follows: $V=\frac{V_{\text{max}}\times C}{K_{\text{m}}+C}$ , where *V* is the uptake rate [in micrograms per gram root dry weight (DW) per hour], *K*_m_ represents the Michaelis constant (in micromoles per liter), *V*_max_ denotes the maximum uptake rate (in micrograms per gram root DW per hour), and *C* is the substrate concentration (in micromoles per liter).

All results were presented as the mean ± SE (*n* = 3). Data were processed using SPSS 20 software using ANOVA and Duncan’s test for multiple comparisons of means between treatments (*p* < 0.05). Analytical data were plotted using OriginPro 2021.

## 3 Results

### 3.1 Characterization of APS-SeNPs

**Figure 1:**
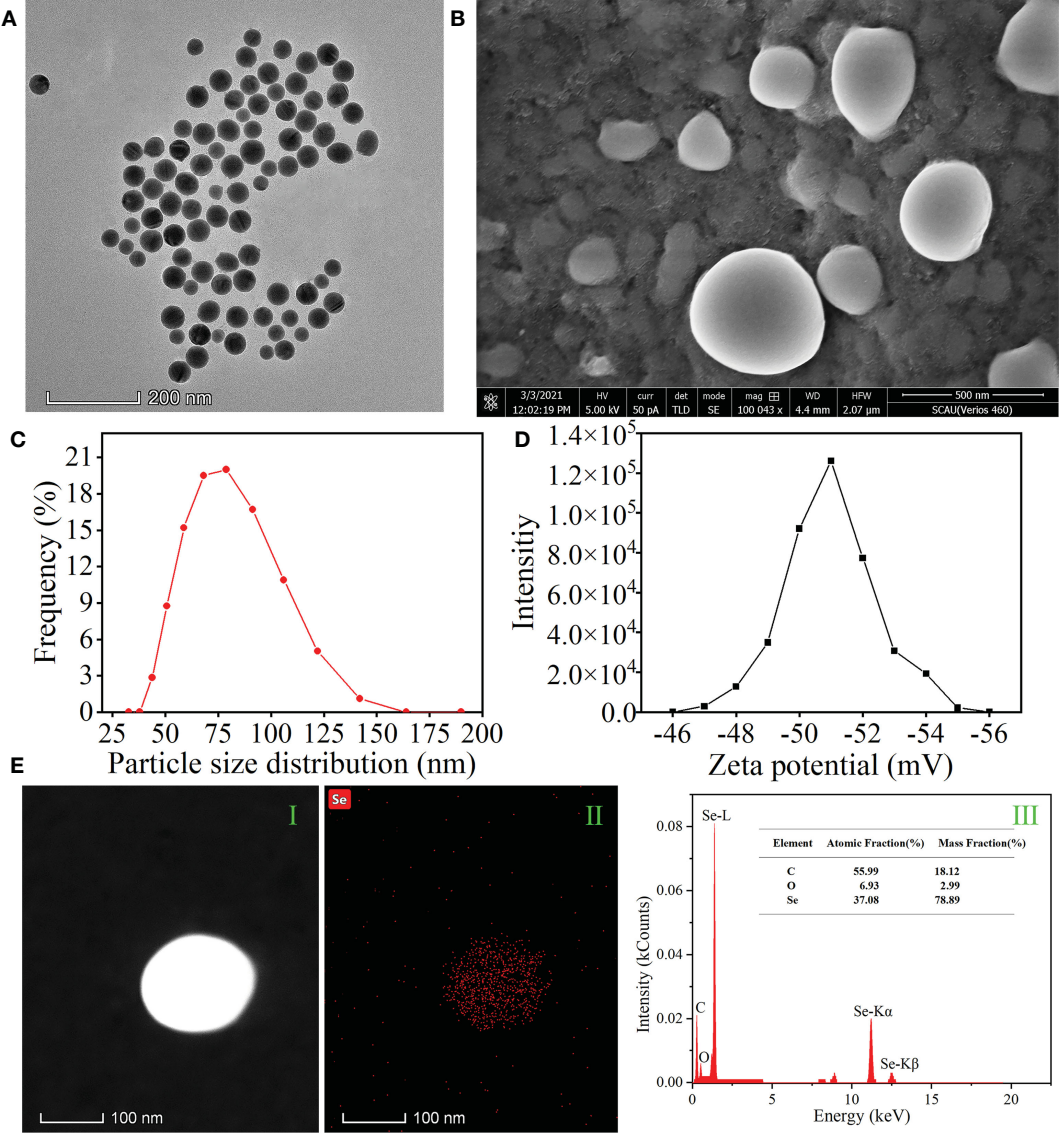
Characterization of algal polysaccharides–selenium nanoparticles (APS-SeNPs). **(A)** Transmission electron microscopy (TEM) images. **(B)** Scanning electron microscopy (SEM) images. **(C)** Particle size distribution. **(D)** Zeta potential. **(E)** TEM/energy-dispersive X-ray spectroscopy [HAADF-TEM (I), X-ray fluorescence image of Se (II), and TEM-EDS (III)].

The microstructure, element distribution, size distribution, and zeta potential of APS-SeNPs were observed. The morphology and the size of APS-SeNPs were characterized using SEM and TEM (**Figures 1A, B**, respectively). The results showed that the synthesized APS-SeNPs appeared as well-dispersed spherical particles with an average diameter of 80 nm. The average particle size was 78.38 nm, while the zeta potential was −46.82 mV as measured by a laser particle size analyzer, indicating good stability (**Figures 1C, D** respectively). The surface element composition of the APS-SeNPs was characterized with TEM-EDS, which showed that Se was distributed on the nanoparticles and accounted for 37.08%. There were specific Se uptake peaks at 1.38 keV (peak Se-L), 11.28 keV (peak Se-Kα), and 12.51 keV (peak Se-Kβ). Furthermore, the synthesized APS-SeNPs also mainly contained C (55.99%) and O (6.93%) elements, indicating that they were successfully **Figures 1E**.

### 3.2 Uptake characteristics of APS-SeNPs by rice roots

**Figure 2:**
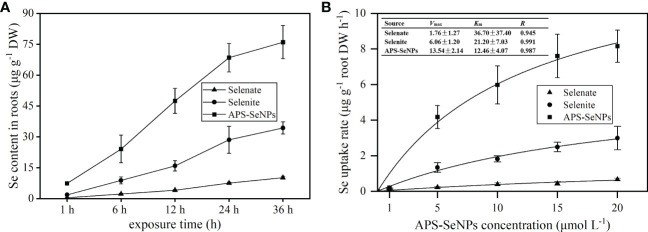
Time- **(A)** and concentration-dependent **(B)** kinetics of algal polysaccharides–selenium nanoparticles (APS-SeNPs), selenite, and selenate.

To evaluate the uptake capacity of rice roots for APS-SeNPs, selenite, or selenate by rice roots, the time- and concentration-dependent kinetics (**Figures 2A, B**) of Se uptake was studied in a hydroponic experiment. **Figure 2A** shows that the uptake of APS-SeNPs, selenite, or selenate by rice roots increased with time. APS-SeNPs reached a plateau at 24 h, while selenite or selenate did not reach a plateau during the experiment. However, the root Se content of the APS-SeNP treatment was significantly higher than that of the selenite or selenate treatment at the same treatment time. The concentration uptake kinetics for APS-SeNPs, selenite, and selenate fitted the Michaelis–Menten equation (**Figure 2B**). It was found that the Se uptake into rice roots increased with the external Se concentration. The *V*_max_ of the influx of APS-SeNPs was 7.69- and 2.23-fold higher than that of selenate or selenite, indicating that rice roots had great uptake potential for APS-SeNPs. Furthermore, the *K*_m_ value of APS-SeNPs was significantly lower than that of selenite or selenate, indicating that rice roots also had a higher affinity for APS-SeNPs than for selenite and selenate.

### 3.3 Uptake method of APS-SeNPs in rice roots

#### 3.3.1 Effect of inhibitors on the uptake of different concentrations of APS-SeNPs by rice

**Figure 3:**
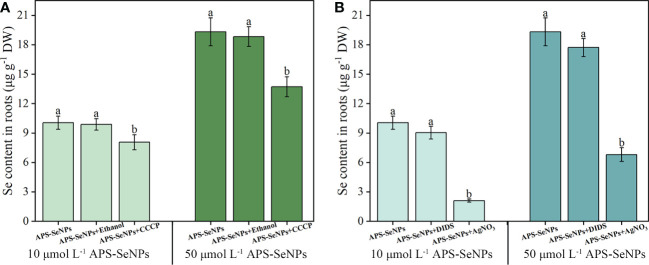
Effect of the metabolic inhibitor CCCP (carbonyl cyanide 3-chlorophenylhydrazone) **(A)** and the anionic inhibitor DIDS (4,4-diisothiocyanatostilbene-2,2-disulfonic acid disodium salt hydrate) and aquaporin inhibitor AgNO_3_ **(B)** on the influx of different concentrations of algal polysaccharide–selenium nanoparticle (APS-SeNP) into rice roots. Data are presented as the mean ± SE (*n* = 4). *Different letters* indicate significant differences between treatments at *p* < 0.05 according to Duncan’s test.

To determine whether APS-SeNPs enter rice roots through passive diffusion or active transport, which consumes energy, an experiment based on the literature was performed to investigate the effects of a metabolic inhibitor (CCCP), an aquaporin inhibitor (AgNO_3_), and an anionic inhibitor (DIDS) on the uptake of different concentrations APS-SeNPs by rice. Compared with the control, the addition of CCCP significantly reduced the uptake of APS-SeNPs by 19.83%–29.03%, but the effect was greater at 50 μmol L^−1^ (**Figure 3A**). Similarly, AgNO_3_ also affected the uptake of APS-SeNPs, which was reduced by 64.81%–79.09%. DIDS and ethanol had no significant effect on the uptake (**Figure 3B**). These results indicate that the uptake of APS-SeNPs is dependent on energy consumption under certain conditions.

#### 3.3.2 Effect of phosphorus/sulfur nutrition on the APS-SeNP uptake of rice

**Figure 4:**
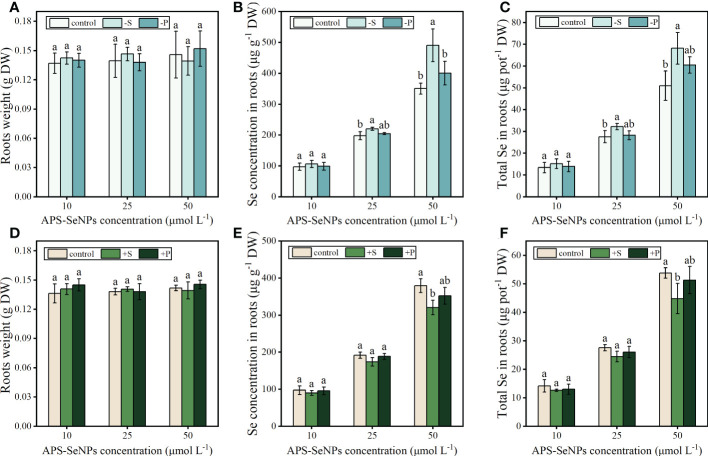
Effect of phosphorus (P) and sulfur (S) deficiency **(A–C)** or P and S supplementation **(D–F)** on the root weight and the uptake and accumulation of APS-SeNPs in rice. Data are presented as the mean ± SE (*n* = 4).Different letters indicate significant differences between treatments at *p* < 0.05 according to Duncan’s test.

To explore the effects of P/S nutrition on the uptake of APS-SeNPs by rice, firstly, the rice roots were pretreated with P- and S-deficient or with P- and S-supplemented [1 mM KH_2_PO_4_ and 1 mM (NH_4_)_2_SO, respectively] solutions and then treated with APS-SeNPs in a normal nutrient solution. Due to the short treatment time, the effect of P or S on the fresh weight of rice root was not significant (**Figures 4A, D**). However, the S- or P-deficient pretreatment promoted the uptake and accumulation of APS-SeNPs in rice roots, in which the accumulation of Se was 1.13–1.34 or 1.03–1.19 times that of the control, separately. In contrast, the S- or P-supplemented pretreatment decreased the uptake and accumulation of APS-SeNPs in rice roots. Furthermore, treatment with the higher concentration of Se showed the more obvious effects of P/S on the uptake of APS-SeNPs by rice, but that of S was more sensitive (**Figures 4B, C, E, F**). These results manifest that S nutrition significantly affected the uptake of APS-SeNPs by rice roots.

#### 3.3.3 Expression of the genes related to the uptake of APS-SeNPs by rice roots

**Figure 5:**
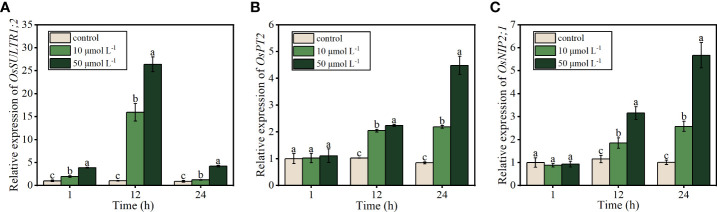
Relative expression of *OsSULTR1;2* **(A)**, *OsPT2* **(B)**, and *OsNIP2;1* **(C)** in relation to the uptake of algal polysaccharides–selenium nanoparticles (APS-SeNPs) in rice roots. Data are presented as the mean ± SE (*n* = 4). *Different letters* indicate significant differences between treatments at *p* < 0.05 according to Duncan’s test.

To understand the channels through which APS-SeNPs enter rice roots and the molecular mechanism of APS-SeNPs being efficiently absorbed by the roots, real-time quantitative PCR (RT-qPCR) was performed to investigate the expression of several genes related to Se uptake. The expression of *OsSULTR1;2*, which encodes the sulfate transporter, increased, followed by a decrease in response to treatment with APS-SeNPs from 1 to 24 h, with the highest expression detected at 12 h of Se treatment. Furthermore, the upregulation level of 50 μmol L^−1^ APS-SeNP was 10 μmol L^−1^, which was 1.66–3.46 times at the same time period (**Figure 5A**). Compared with the control, the genes encoding the phosphate transporter *OsPT2* and the silica transporter *OsNIP2;1* were unaffected at 1 h of treatment with APS-SeNPs. However, the expression levels of *OsPT2* and *OsNIP2;1* increased with time at 12–24 h of treatment in rice roots, with Se treatment at 50 μmol L^−1^ resulting in significantly higher levels compared to that at 10 μmol L^−1^ (**Figures 5B, C**).

### 3.4 Transport and distribution of APS-SeNPs in rice

#### 3.4.1 Se distribution in rice after treatment with different Se sources under hydroponics

**Figure 6:**
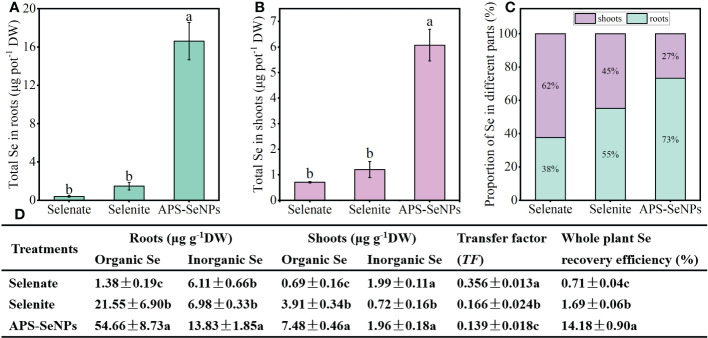
**(A, B)** Accumulation of selenium (Se) in the roots **(A)** and shoots **(B)**. **(C)** Distribution of Se in rice. **(D)** Contents of organic and inorganic Se in the roots/shoots, translocation, and plant Se recovery efficiency under different Se treatments. Data are presented as the mean ± SE (*n* = 4). *Different letters* indicate significant differences between treatments at *p* < 0.05 according to Duncan’s test.

To compare the uptake and transport capacity of rice for the different Se sources, 10 μmol L^−1^ APS-SeNPS, selenite, or selenate was added to the nutrient solution for treatment for 24 h. **Figure 6** shows the distinct Se accumulation in the same parts of rice with respect to the different Se treatments. The Se accumulation in the rice roots and shoots with APS-SeNP treatment was significantly higher than that with selenite or selenate treatment, which was mainly in the form of organic Se. The transfer factor (TF) can be used to assess the ability of Se to transfer from the roots to the shoots. The TF was the highest in the selenate treatment, where 62% of the Se was transported to the shoots mainly in the inorganic form. On the other hand, the TF in the selenite or APS-SeNP treatment was lower, and Se was mainly accumulated in its organic form in the roots. Furthermore, the Se recovery efficiency of the whole plant with different Se treatments showed the following order: APS-SeNPs > selenite > selenate.

**Figure 7:**
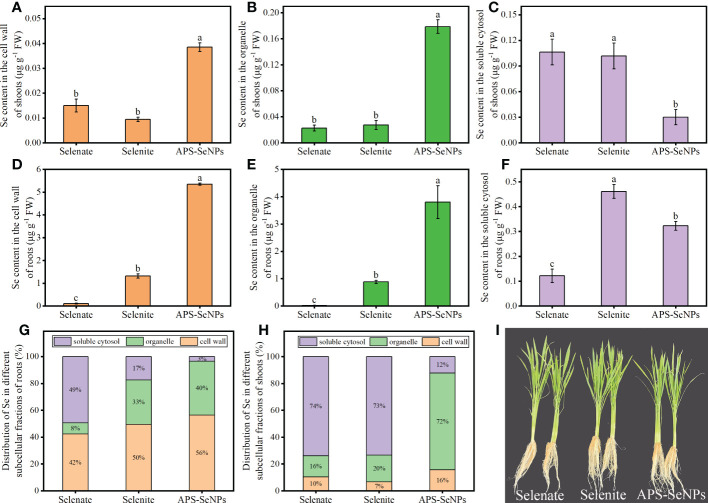
**(A–F)** Selenium (Se) contents of the cell wall, organelle, and soluble cytosol in rice shoots **(A–C)** and roots **(D–F)** under different Se treatments. **(G, H)** Se distribution in the subcellular fractions of roots **(G)** and shoots **(H)**. Data are presented as the mean ± SE (*n* = 4). *Different letters* indicate significant differences between treatments at *p* < 0.05 according to Duncan’s test.

The Se contents in the subcellular fractions of rice roots and shoots were determined in this experiment. The Se content in the shoot subcellular fraction of rice varied under different Se treatments. The Se contents of the cell wall (F1) and the organelle (F2) in rice shoots treated with APS-SeNPs were significantly higher than those in rice shoots treated with selenite or selenate, with Se mainly distributed in the organelle. The Se in the shoots resulting from selenite or selenate treatment was mainly distributed in the soluble cytosol (F3), and its content was increased by 238.81% or 254.06% compared to that under APS-SeNP treatment, respectively (**Figures 7A–C, H**). In addition, the Se content in the subcellular fraction of rice roots also varied between different Se treatments. The Se contents in the cell wall (F1), organelle (F2), and soluble cytosol (F3) with APS-SeNP or selenite treatment were higher than those with selenate treatment. Under selenite or APS-SeNP treatment, the distribution of Se in the subcellular fraction of roots was in the order cell wall > organelle > soluble cytosol. However, the Se distribution in the roots with selenate treatment was different, which showed the order soluble cytosol > cell wall > organelle (**Figures 7D–F, G**).

#### 3.4.2 Distribution of Se in rice under soil cultivation

The Se content of each part of rice was measured under soil cultivation, with the results shown in **Table 2**. Se application significantly enhanced the Se contents of the roots, stems, leaves, and grains, but the distribution of Se was different as a result of the different Se treatments. The Se content of the leaves was higher with selenate treatment, while that of the roots was higher with selenite or APS-SeNP treatment. Notably, the Se contents of rice roots, stems, and grains treated with APS-SeNPs were significantly higher than those treated with selenite or selenate, which were 1.36–1.84, 1.06–1.68, 1.47–1.61, respectively, except for the leaves. These results indicate a strong transport capacity from the leaves to the grains resulting from APS-SeNPs, and the aboveground Se mainly accumulated in the grain, thus achieving an efficient Se enrichment effect.

**Table 2:** Selenium (Se) contents of the different parts of rice under different Se treatments.

Treatment	Root (μg g^−1^ DW)	Stem (μg g^−1^ DW)	Leaf (μg g^−1^ DW)	Grain (μg g^−1^ DW)
CK Selenate	0.72 ± 0.04 d 1.28 ± 0.17 c	0.40 ± 0.06 c 1.32 ± 0.02 a	0.45 ± 0.04 d 1.54 ± 0.12 a	0.13 ± 0.02 c 0.30 ± 0.02 b
Selenite	1.74 ± 0.09 b	0.83 ± 0.06 b	1.12 ± 0.19 b	0.28 ± 0.03 b
APS-SeNPs	2.36 ± 0.14 a	1.40 ± 0.12 a	0.77 ± 0.06 c	0.44 ± 0.05 a

Data are presented as the mean ± SE (n = 4). Different letters after values within the same column indicate significant difference among treatments (p < 0.05).

CK, control treatment; DW, dry weight; APS-SeNPs, algal polysaccharides–selenium nanoparticles

**Figure 8:**
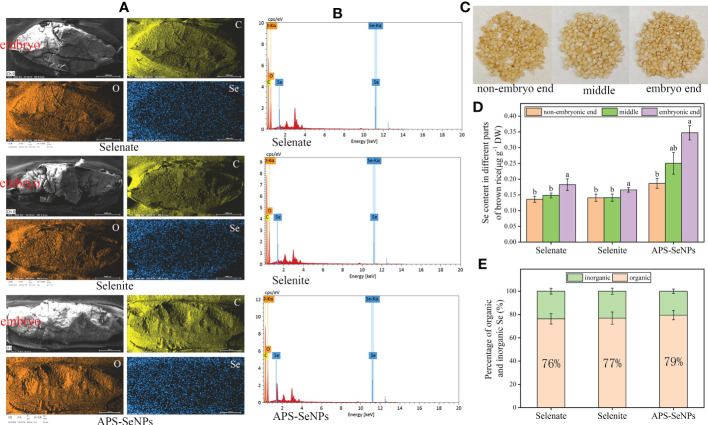
Grain selenium (Se) distribution under different Se treatments. **(A)** X-ray fluorescence image. **(B)** Transmission electron microscopy with energy-dispersive X-ray spectroscopy (TEM-EDS). **(C)** Physical picture of each part of the grain. **(D)** Se contents of the different parts of rice grain. **(E)** Organic and inorganic ratios under different treatments. Data are presented as the mean ± SE (*n* = 4). *Different letters* indicate significant differences between treatments at *p* < 0.05 according to Duncan’s test.

The ultimate goal of Se biofortification in rice is to increase the Se content in the grains. However, the content and distribution of Se in rice grains differed under the different Se treatments. In this study, the brown rice was cut into three equal sections, namely, the embryo end, middle and non-embryo end (**Figure 8C**). **Figures 8B, E** reveal that the Se content in brown rice (without husk) was distinct with respect to the different treatments. The Se content in brown rice with APS-SeNP treatment was the highest, and 79% was present in organic form. In addition, the Se distribution in rice grains was heterogeneous. **Figures 8A, D** show that the distributions of C and O were not significantly different among the different treatments, while Se was densely distributed in the embryo end and sparsely in the non-embryo end, with the Se content at the embryo end being 1.18–1.87 times than that at the non-embryo end. Interestingly, the Se contents in the different parts of rice following APS-SeNP treatment were higher than those after selenate or selenite treatment. Data for the control treatment were not included as the Se content at the non-embryo end of the control treatment could not be measured.

## 4 Discussion

### 4.1 Uptake mechanism of APS-SeNPs

SeNPs are bright red particles with a diameter between 5 and 200 nm that can be synthesized by physical, chemical, and biological methods (Kumar and Prasad, 2021). In this paper, APS-SeNPs were synthesized using APS as a stabilizer, while ascorbic acid was used as a reducing agent for sodium selenite. The concentration uptake kinetics was consistent with the Michaelis–Menten equation (**Figure 2**), which indicated that the uptake of APS-SeNPs in rice roots was influenced by the transporter protein. A previous study showed that AgNO_3_ inhibited the entry of APS-SeNPs into rice by 60.4%, while CCCP decreased the uptake of APS-SeNPs by 16.2% (Wang et al. 2020a). AgNO_3_ is an inhibitor of aquaporins that inhibits the sulfhydryl reaction of silver with cysteine and histidine, leading to the gating of targeted aquaporins (Niemietz and Tyerman, 2002). CCCP is an oxidative phosphorylation and proton carrier uncoupling agent that causes the proton dynamics to diffuse across the membrane, thereby reducing the proper functioning of ATP synthase (Zhang et al., 2014). Based on the literature, this paper explored the response of different concentrations of APS-SeNPs to inhibitors. The results showed that AgNO_3_ and CCCP decreased the uptake of APS-SeNPs by rice, while DIDS did not have a significant effect (**Figure 3**), further demonstrating that the uptake process of APS-SeNPs is dependent on energy consumption. The results of this study, which showed AgNO_3_ inhibiting the uptake of APS-SeNPs in rice by more than 64.81% and CCCP inhibiting it by less than 29.03% (**Figure 3**), can be explained by the entry of most APS-SeNPs into rice roots through aquaporin; however, due to the unique physiological properties of APS-SeNPs, in addition to aquaporin, some APS-SeNPs may also be transported along the channels of APS, among others (such as sulfate transporters), against the electrochemical gradient into the plasma membrane of root cells. However, the specific channels need to be further investigated.

Previous studies have demonstrated that selenate and selenite enter plant roots *via* sulfate transporters and *via* phosphate or silicate transporters, respectively (Zhao et al., 2010a; Zhang et al., 2014; Maruyama-Nakashita, 2016; Zhang et al., 2020b). In plant roots, a deficiency of S increased the selenate uptake and upregulated the expression of the sulfate transporter gene (El Mehdawi et al., 2018), while a deficiency of P increased the uptake of selenite and upregulated the expression of the phosphate transporter gene (Zhang et al., 2014; Li et al., 2019). However, it is unclear whether the mechanism of APS-SeNP uptake by plant roots is related to the sulfate, phosphate, or silicate transporter. In this study, S deficiency promoted the uptake of APS-SeNPs, whereas S supplementation decreased the APS-SeNP uptake in rice roots (**Figure 4**) and upregulated the expression of the sulfate transporter (*OsSULTR1;1*) with APS-SeNP treatment (**Figure 5**). This indicates that S plays an important role in the uptake of APS-SeNPs by rice roots. In addition, the silicon influx transporter (*OsNIP2;1*) is a member of the nodulin 26-like intrinsic membrane protein subfamily of aquaporins that is expressed in the roots (Zhao et al., 2010b). *OsPT2* is a phosphate transporter responsible for the transport of inorganic phosphate (Pi) in rice (Yang et al., 2020). Teixeira et al. (2021) showed that Se enhanced the expression of *OsPT2* and *OsNIP2;1* in the roots. This finding was similar to that in this paper, which showed that the expression of *OsPT2* and *OsNIP2;1* in rice roots increased with time after 12 h treatment with APS-SeNPs (**Figure 5**). This further indicated that multiple genes are involved in the regulation of the uptake of APS-SeNPs by rice.

### 4.2 Transport and distribution of Se in different parts of rice

Plants use different uptake mechanisms in response to different Se treatments. The uptake capacity of organic Se is higher than that of inorganic Se, with the order being selenomethionine > selenomethionine oxide > selenite > selenate (Wang et al., 2022), which is related to the plant genotype, environmental conditions, and the affinity of the transport proteins in plant root epidermal cell membranes for Se. In the current study, the uptake rate of APS-SeNPs was significantly higher than that of selenate and selenite (**Figure 2**), which is in contrast to the findings of Hu et al. (2018). This discrepancy may be related to the stabilizer used in the preparation of APS-SeNPs, which has good cellular affinity and can be rapidly absorbed by plant roots. On the other hand, algal polysaccharide-modified SeNPs have a smaller size and stronger stabilization and dispersion than bare SeNPs (Wang et al., 2021a), thus allowing more APS-SeNPs to be taken up by plant roots. However, the exact reason for this needs to be further investigated.

The cumulative distribution of Se in different plant parts is closely related to its transport. Studies have shown that selenate is extremely mobile in the xylem, while Se is translocated directly from the roots to the shoots (Li et al., 2008). A similar result was found in tomato (Wang et al., 2019a). While selenite or SeNPs were absorbed by plant roots and then converted to selenomethionine or other organic forms (Zhang et al., 2019), their TF were low, resulting in most of the Se being trapped in the roots. In this study, after 24 h of exposure, the TF was higher with selenate treatment, with most of the Se being transported aboveground; in contrast, the TF was lower under selenite or APS-SeNP treatment, allowing Se to mainly accumulate in the roots (**Figure 6**). This is consistent with previous research results (Li et al., 2008; Wang et al., 2020a). Interestingly, however, the Se contents of all parts of rice plants treated with APS-SeNPs were significantly greater than those of selenite or selenate treatment, which may be related to the transport capacity of Se and the expression of the transport genes. Furthermore, the results of the soil pot experiments also showed that APS-SeNP or selenite treatment resulted in Se being mainly distributed in the roots, while Se was focused in the leaves under selenate treatment (**Table 2**). These results were similar to those of Wang et al., (2021a). The distribution of Se in the subcellular fraction of roots can also affect its accumulation in plants. The cell wall is the major site for the sequestration of toxic elements (Yu et al., 2019). It is mechanically rigid and acts as a barrier to the movement of heavy metals or metal-like ions across the cell membrane, thereby inhibiting their translocation (Hall, 2002). This study showed that Se was mainly distributed in the cell wall of roots under selenite or APS-SeNP treatment, which restricted its transport; however, most of the Se after selenate treatment was distributed in the soluble cytosol, which allowed unimpeded translocation from the roots to the shoots (**Figure 7**).

The ultimate goal of Se biofortification is to enhance the organic Se content of the edible parts of plants (Premarathna et al., 2012). Previous studies have shown that the percentage of organic Se in cereal seeds was 50%–80% (Eiche et al., 2015; Gong et al., 2018). In this study, the percentage of organic Se in brown rice treated with different concentrations of Se were all more than 75%, in the following order: APS-SeNPs > selenite ≈ selenate (**Figure 8**). The organic Se in brown rice resulting from selenate treatment was two fold greater under selenite treatment, according to Deng et al. (2017). This suggests that the test conditions and plant genotypes have a strong influence on the effectiveness of Se enrichment in crops. During rice grain filling, Se is transported from the leaf to the grain, and the endosperm is the main storage of nutrients. Se is mainly located in the longitudinal direction of the endosperm (de Lima Lessa et al., 2020). However, Zhang et al. (2020a) showed that the distribution of Se in the grain was related to the crop variety, with the high-Se rice cultivar having a high storage capacity, resulting in the uniform distribution of Se, while the Se in the low-Se cultivar was mainly distributed in the embryo end of the grain. In this study, the Se content at the embryo end was significantly higher than that at the non-embryo end in the grains of brown rice (**Figure 8**). This further indicated that the spatial distribution of Se in rice grains is related to the plant genotype.

## 5 Conclusions

In this study, we preliminarily determined the uptake pattern and distribution characteristics of APS-SeNPs in rice. The concentration-dependent kinetics of the uptake of APS-SeNPs in rice roots fitted the Michaelis–Menten equation, which was sensitive to aquaporin and metabolic inhibitors. Sulfur starvation promoted the uptake of APS-SeNPs, while phosphorus starvation had no significant effect, and treatment with APS-SeNPs enhanced the expression of *OsSULTR1;2* in rice roots. The chemical form of Se affected its uptake and distribution in rice. Compared with selenate or selenite treatment, APS-SeNP treatment significantly increased the Se contents of the roots, stems, and grains, as well as the plant Se recovery efficiency. Most of the Se in the roots of plants treated with APS-SeNPs was distributed in the cell wall and was in organic form, while 44.31% of the Se in the rice grains was distributed in the embryo end and was also in organic form. Therefore, based on these results, it can be concluded that APS-SeNPs constitute a potential new type of Se-rich fertilizer that can be applied in agriculture. It is anticipated that the findings of this study would provide a theoretical basis for the uptake and distribution of APS-SeNPs in rice plants.

## Data availability statement

The original contributions presented in the study are included in the article/supplementary material. Further inquiries can be directed to the corresponding author.

## Author contributions

HS, CW, and CY: conceived and designed the experiments. CY: performed the experiments and wrote the manuscript. HS, ZK, and ZL: reviewed and edited the manuscript. SD: provided technical support for the gene expression study. All authors contributed to the article and approved the submitted version.
